# Breaking the in-coupling efficiency limit in waveguide-based AR displays with polarization volume gratings

**DOI:** 10.1038/s41377-024-01537-8

**Published:** 2024-08-12

**Authors:** Yuqiang Ding, Yuchen Gu, Qian Yang, Zhiyong Yang, Yuge Huang, Yishi Weng, Yuning Zhang, Shin-Tson Wu

**Affiliations:** 1 College of Optics and Photonics, University of Central Florida, Orlando, FL 32816 USA; 2 Joint International Research Laboratory of Information Display and Visualization, Southeast University, Nanjing, 210096 China; 3 Meta Reality Labs Research, 9845 Willows Road NE, Redmond, WA 98052 USA

**Keywords:** Displays, Imaging and sensing

## Abstract

Augmented reality (AR) displays, heralded as the next-generation platform for spatial computing, metaverse, and digital twins, empower users to perceive digital images overlaid with real-world environment, fostering a deeper level of human-digital interactions. With the rapid evolution of couplers, waveguide-based AR displays have streamlined the entire system, boasting a slim form factor and high optical performance. However, challenges persist in the waveguide combiner, including low optical efficiency and poor image uniformity, significantly hindering the long-term usage and user experience. In this paper, we first analyze the root causes of the low optical efficiency and poor uniformity in waveguide-based AR displays. We then discover and elucidate an anomalous polarization conversion phenomenon inherent to polarization volume gratings (PVGs) when the incident light direction does not satisfy the Bragg condition. This new property is effectively leveraged to circumvent the tradeoff between in-coupling efficiency and eyebox uniformity. Through feasibility demonstration experiments, we measure the light leakage in multiple PVGs with varying thicknesses using a laser source and a liquid-crystal-on-silicon light engine. The experiment corroborates the polarization conversion phenomenon, and the results align with simulation well. To explore the potential of such a polarization conversion phenomenon further, we design and simulate a waveguide display with a 50° field of view. Through achieving first-order polarization conversion in a PVG, the in-coupling efficiency and uniformity are improved by 2 times and 2.3 times, respectively, compared to conventional couplers. This groundbreaking discovery holds immense potential for revolutionizing next-generation waveguide-based AR displays, promising a higher efficiency and superior image uniformity.

## Abstract (web summary)

An anomalous polarization conversion in polarization volume gratings is discovered to break the in-coupling efficiency limit in waveguide displays, which holds immense potential for revolutionizing next-generation augmented reality glasses.

## Graphical abstract

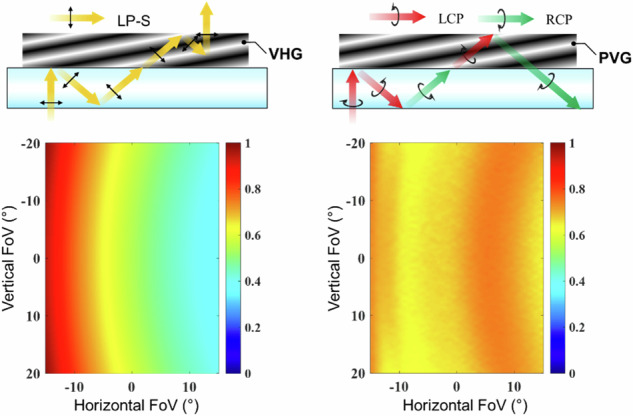

## Introduction

After decades of device innovation and vibrant advances in microdisplay technologies, ultra-compact imaging optics, and high-speed digital processors, augmented reality (AR) has evolved from a futuristic concept to a tangible and pervasive technology. By seamlessly blending the projected virtual content with real-world scenes, AR enhances our perception and interaction with environment, opening exciting possibilities for metaverse, digital twins, and spatial computing. AR displays have enabled widespread applications in smart education and training, smart healthcare, navigation and wayfinding, gaming and entertainment, and smart manufacturing, just to name a few.

Since its primitive conception in the 1990s, AR has made significant strides, particularly with the emergence and development of waveguide-based AR displays. These displays enable wearable systems to be lightweight and have a slim form factor while maintaining high optical performance. Furthermore, the rapid development of couplers, including partial reflective mirrors, surface relief gratings (SRGs), volume holographic gratings, polarization volume gratings (PVGs), metasurfaces, etc., has dramatically improved the optical performance of AR displays over the past few decades^1–5^.

While waveguide displays have dramatically reduced the form factor, the low efficiency of optical combiners, particularly the diffractive waveguide combiners, remains a major concern^6^. In the era of modern wireless near-eye displays powered by batteries, such a low optical efficiency imposes a significant challenge, ultimately limiting the continuous operation time.

**Fig. 1:**
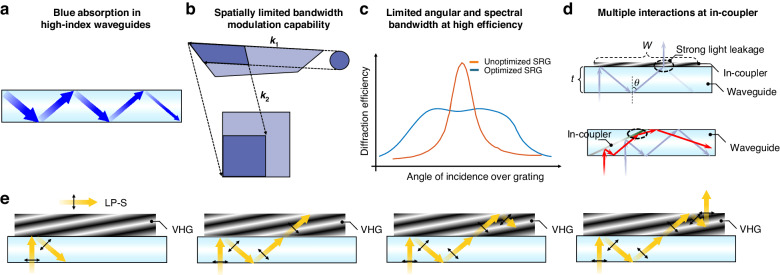
Mechanisms of light loss in a waveguide-based AR display. **a** Blue light absorption during propagation in a high-index waveguide substrate. **b** Effective pupil expansion process for a certain field angle in a traditional 2D exit pupil expansion (EPE). **c** Angular response for unoptimized and optimized SRGs. **d** Light loss due to multiple interactions at the diffractive in-coupler^1^ and geometric in-coupler. **e** Multiple interaction processes between the incident beam and the intensity-type VHG in-coupler

The low optical efficiency primarily stems from four aspects, all related to the nonuniformity issues, such as color nonuniformity, FoV nonuniformity, and eyebox nonuniformity^1,2,6^. The first major optical loss originates from the absorption and scattering of a high-index waveguide substrate^7^, as shown in Fig. 1a. These substrates are typically used in a full-color diffractive waveguide display to enlarge its FoV. For instance, a 10 mm-thick high-index waveguide substrate from AGC Inc. exhibits a transmittance of about 95% in the blue spectral region. Due to multiple total internal reflections, the effective propagation distance in a 1 mm-thick waveguide can be as large as 50 mm. As a result, about 23% of the blue light is absorbed. Such a noticeable optical loss not only lowers the overall waveguide efficiency but also degrades the color uniformity. To obtain a balanced white at exit pupil, say D65, we must increase the power of the blue channel. However, with active development of waveguide materials, and fabrication and purification processes, this absorption/scattering loss could be gradually mitigated, becoming less significant over time.

The second major source of optical loss occurs during the pupil expansion process. As illustrated in Fig. 1b, a significant portion of light is wasted due to the spatially limited bandwidth modulation capability of the folding couplers and out-couplers^2^. Specifically, the effective pupil expansion process for a given field angle utilizes only a small portion of the folding coupler and out-coupler. If these components fail to accurately control the angular and spectral response spatially, any exit pupil expansion beyond the effective area will lead to light wasting.

The third major optical loss mechanism is the limited angular and spectral bandwidth of the couplers in the high diffraction efficiency region^1,2^. As the FoV increases, the coupler’s bandwidth may not be sufficient to maintain a good uniformity. To improve uniformity, lower efficiency couplers are often compromised to achieve a broader bandwidth^8–10^, as illustrated in Fig. 1c. Importantly, a larger FoV exacerbates energy loss during this process. For instance, during Photonics West 2024, Applied Materials demonstrated two SRG-based full-color waveguide displays with different FoVs. The display with a 20° FoV has an efficiency of 4500 nits lm^−1^ (~10%), while the display with a 30° FoV only achieves 1300 nits lm^−1^ (~3%).

As depicted in Fig. 1d, the fourth major cause of low-efficiency results from multiple interactions at the in-couplers^11,12^. Significant light leakage occurs at the in-coupler to maintain a good eyebox uniformity, even when the in-coupler is a fully reflective mirror or a grating with 100% diffraction efficiency. For instance, when a traditional diffractive grating, such as an SRG or intensity-type VHG, is used as an in-coupler, the waveguide combiner experiences substantial optical loss due to multiple interactions between the incident light and the in-coupler, especially at extreme FoV angles. This light leakage not only deteriorates the uniformity across the entire FoV but also reduces the ambient contrast ratio of the virtual images. A larger FoV exacerbates this loss. It is also worth noting that a similar process occurs in geometric waveguide combiners; however, in this case, the second or multiple interactions merely alter the propagation direction, as illustrated by the red lines in Fig. 1d, leading to stray light^13^.

Light leakage at the in-coupler is a longstanding issue over the past few decades^1,2,11,12,14^, yet no good solution can completely overcome this tough problem because it is fundamentally unavoidable with conventional in-couplers, including SRG, intensity-type VHG, and even metasurface devices. Recent studies^12^ have indicated that the second interaction mirrors the symmetric process of the first interaction, implying that almost all the light experiencing the second interaction will either be coupled out of the waveguide or change its propagation direction if the diffraction efficiency is 100%. As further examined in conventional couplers (Fig. 1e), the second interaction is essentially a reverse process of the first interaction. This is primarily because conventional couplers are made of isotropic materials, leading to no polarization change during the interaction process.

As depicted in Fig. 1d, if the size ($W$) of the in-coupler is greater than the total internal reflection (TIR) propagation distance $d=2t* \tan \left(\theta \right)$, where *t* represents the waveguide thickness and $\theta$ denotes the TIR angle inside the waveguide, then the in-coupling light will interact with the in-coupling grating two or more times. In practical terms, achieving a continuous eyebox necessitates that $W$ > $d$. Otherwise, users may not perceive digital information in certain regions within the eyebox. Thus, even if the width of the in-coupler can be reduced by increasing the collecting power of the collimation lenses or shrinking the emission cone of the microdisplay panels, the TIR angle $\theta$ or waveguide thickness $t$ must be decreased accordingly to maintain a good continuity throughout the eyebox. Consequently, efficiency loss and poor uniformity throughout the FoV persist as significant challenges. For example, in a single-color waveguide display with a $70^{\circ}$ (45°(H) × 55°(V)) FoV in a substrate with an index *n* = 2.0, over 70% of the incident light is lost due to the multiple interactions at the in-coupling process for extreme field angles, resulting in a 70% drop in FoV uniformity. To boost the FoV uniformity, the overall in-coupling efficiency is compromised. For a full-color, 30°(24°(H) × 18°(V)) FoV waveguide display using a *n* = 2 substrate, the in-coupling efficiency loss for the blue and red colors at extreme field angles exceeds 73% and 51.5%, respectively, causing a drop in color uniformity of around 45%. To balance both color uniformity and FoV uniformity, the overall efficiency is also compromised. The calculation method for these examples will be discussed in the following section. Therefore, finding a solution to circumvent the tradeoff between in-coupling efficiency, uniformity throughout the FoV, and eyebox continuity is urgently needed.

In this paper, we present the discovery of an anomalous polarization conversion phenomenon in the PVGs. This phenomenon offers an intuitive solution to the abovementioned issue for achieving a high and uniform in-coupling efficiency throughout the entire FoV while maintaining continuous eyebox functionality. To prove concept, preliminary experiments are conducted to validate this polarization conversion process. The experimental results closely align with the Rigorous Coupled-Wave Analysis (RCWA) simulation. Moreover, the in-coupling efficiency limit of a 50° FoV waveguide-based AR display system is enhanced by two times with the first-order polarization conversion in a PVG, compared to conventional couplers. Concurrently, the uniformity throughout the FoV is also improved by 2.3 times. Furthermore, by combining an additional polarization compensation film at the in-coupler, nearly all light can be coupled into the waveguides.

## Results

**Fig. 2:**
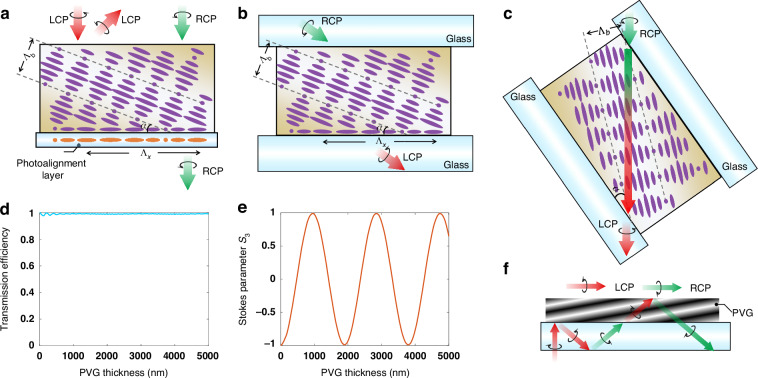
Working principles of PVG as in-coupler in waveguide displays. **a** Slanted structure of PVG, which reflects LCP and transmits RCP. **b** PVG functions as a tilted twisted-nematic (TN) LC waveplate when the incident angle of light approaches the Bragg plane. **c** PVG functions as a tilted TN LC waveplate from the perspective of incident angle by rotating PVG. **d** 0th order transmission efficiency and (**e**) Stokes parameter ${S}_{3}$ of transmitted light varying with the PVG thickness. The birefringence of PVG for simulation is 0.4 (${n}_{e}=2.0,\,{n}_{o}=1.6$); input and output media are both glass with ${n}_{g}=$1.7; horizontal period ${\Lambda }_{x}$ and slanted angle $\alpha$ of the PVG are 407 nm and 23.3°; the incident angle ${\theta }_{{in}}$ and wavelength $\lambda$ are −45° and 532 nm. **f** Schematic of polarization conversion during the interaction between incident light and PVG without considering phase shift induced by Fresnel reflection

The PVG is a polarization-selective holographic optical element that records the polarization information of two interfering beams comprising a right-handed circular polarization (RCP) and a left-handed circular polarization (LCP). As illustrated in Fig. 2a, PVG features a slanted cholesteric liquid crystal (CLC) structure, where the liquid crystal (LC) directors rotate along the helical axis^15–21^. This CLC structure endows PVG with the polarization-selective characteristic of CLC, as depicted in Fig. 2a. It reflects the circular polarization state possessing the same handedness as the helical twist of the CLC while transmitting the opposite component. For example, it diffracts LCP while permitting RCP to pass through. Additionally, the first-order (${R}_{1}$) diffraction efficiency increases with the CLC pitch number and then gradually saturates.

However, here we discover an anomalous phenomenon that deviates from the abovementioned rule. As depicted in Fig. 2b, when the incident angle in the glass substrate approaches the Bragg plane, the Bragg condition does not hold. Consequently, the PVG functions as a waveplate instead of a grating, altering the polarization state of the incident light. For instance, it converts RCP to LCP when the half-wave condition is satisfied. More specifically, from the perspective of the incident light direction, as shown in Fig. 2c, the PVG resembles a tilted twisted-nematic (TN) liquid crystal waveplate^22^ with a very long pitch ($P$),

1 $$P=\frac{2{\Lambda}_{b}}{\sin \left(\frac{\pi}{2}-\alpha -{{\theta}_{in}}\right)}$$

where ${\Lambda }_{b}$ and $\alpha$ represent the Bragg period and the slanted angle of the PVG, ${\theta }_{{in}}$ corresponds to the incident angle in the waveguide substrate. To demonstrate the waveplate behavior, the 0th order transmission efficiency and output polarization state are simulated using the RCWA model^23,24^. As shown in Fig. 2(d, e), nearly all the light transmits through the PVG without changing its propagation direction, regardless of the PVG thickness. Additionally, the polarization state (represented by the Stokes parameter ${S}_{3}$) of the transmitted light oscillates as the PVG thickness increases.

Due to these two superior polarization properties, employing PVG as an in-coupler in waveguide displays can dramatically enhance the in-coupling efficiency and uniformity throughout the FoV, while keeping a good eyebox continuity (or uniformity), in comparison with all other traditional in-couplers and metasurface couplers. Specifically, as illustrated in Fig. 2f, the incident LCP light is deflected into the waveguide substrate during the first interaction and retains its polarization state based on the selectivity rule of PVG. After the first interaction, following TIR, the polarization state of light becomes RCP (the 1st green arrow) due to the reversed propagation direction. During the second interaction with the in-coupler (PVG), the light undergoes polarization conversion without altering its propagation direction, meaning the light turns to LCP if the PVG thickness satisfies the half-wave condition. Subsequently, after another TIR at the top boundary of the PVG, the light becomes RCP, which is then transmitted through the PVG due to its polarization selectivity. Finally, the light can propagate inside the waveguide while maintaining its propagation direction. Consequently, the in-coupling efficiency and uniformity are significantly improved while maintaining a desired eyebox continuity.

However, TIR is accompanied by a non-trivial phase shift as the Fresnel reflection coefficient acquires a non-zero imaginary part^25^. Besides, different polarization states, e.g., $s$ and $p$ lights will be introduced a different phase shift, which depends on the polarization of the incident wave as shown below:

2 $${\delta }_{s}=2\,{\tan }^{-1}\left(\frac{\sqrt{{n}^{2}{\sin }^{2}{\theta }_{{in}}-1}}{n\cos {\theta }_{{in}}}\right)$$

3 $${\delta }_{p}=2\,{\tan }^{-1}\left(\frac{n\sqrt{{n}^{2}{\sin }^{2}{\theta }_{{in}}-1}}{\cos {\theta }_{{in}}}\right)$$

where ${\delta }_{s}$ and ${\delta }_{p}$ represent the additional phase induced by TIR process, $n$ is refractive index of glass substrate, and ${\theta }_{{in}}$ denotes the incident angle. This indicates that circular polarization will no longer be pure after TIR because of the phase difference induced by $s$ and $p$ polarization states. Therefore, the incident light during the second interaction in the above situation is not a pure circular polarization state and the optimal thickness will shift.

To validate the concept, we conducted an experiment using a 532 nm laser source and PVGs with varying thicknesses. We employed reactive mesogen RM257, which possesses a birefringence $\varDelta n$ = 0.162 at ${\lambda }$ = 532 nm, to fabricate the PVGs with different thicknesses. This was achieved by adjusting the concentration and spin coating speed on a waveguide substrate with *n* = 1.57. While other liquid crystal materials are also feasible, they may yield different half-wave conditions if they exhibit a different birefringence. The horizontal period ${\Lambda }_{x}$ was set at 411 nm. To accommodate a central wavelength at 532 nm and normal incidence, the slanted angle $\alpha$ is approximately 27.83°, determined based on the following Bragg equation:

4 $$2{n}_{{eff}}{\Lambda }_{b}\cos ({\theta }_{{in}}+\alpha )={\lambda }_{b}$$

where ${n}_{{eff}}$ represents the effective refractive index of the RM257, ${\Lambda }_{b}$ is the Bragg period, ${\lambda }_{b}$ is the Bragg wavelength, and ${\theta }_{{in}}$ is the incident angle.

**Fig. 3:**
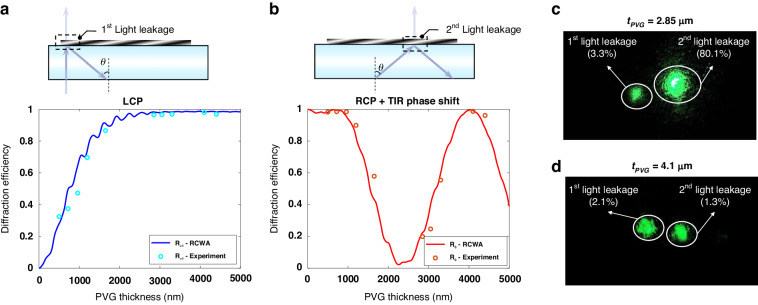
Experimental results of the anomalous polarization conversion in PVG. **a** +1st order ($${R}_{+1})$$ diffraction efficiency changes with thickness during the first interaction for the incident LCP light. **b** 0th order ($${R}_{0}$$) diffraction efficiency changes with thickness during the second interaction for the incident RCP light with an extra TIR phase shift. **c** Light leakage at the PVG as in-coupler for the thickness of 2.85 µm and (**d**) 4.1 µm

Furthermore, Fig. 3 illustrates the experimental results of the diffraction response of PVG at various thicknesses. In Fig. 3a, it is evident that the first-order ($${R}_{1}$$) diffraction efficiency at normal incidence increases with the increased PVG thickness ($${t}_{{PVG}})$$. Simultaneously, using an out-coupling prism, the second interaction (*R*_0_ diffraction order) at a large incidence angle, calculated using $$-{\sin }^{-1}({\lambda }_{b}/{(\Lambda }_{x}* n))$$ in the glass substrate, also depends on the PVG thickness, as depicted in Fig. 3b. This dependency agrees well with the RCWA simulation results. Furthermore, the polarization state of the light leakage during the second interaction remains consistent (LCP). This phenomenon further supports our previous analysis. Additionally, the light leakage in two PVGs with different thicknesses was captured by a camera, as shown in Fig. 3c and d. The left and right beams represent light leakage at the first and second interactions, respectively. It is evident that light leakage is strong when $${t}_{{PVG}}=2.85\,\mu {\rm{m}}$$ but much weaker at $${t}_{{PVG}}=4.1\,\mu {\rm{m}}$$.

**Fig. 4:**
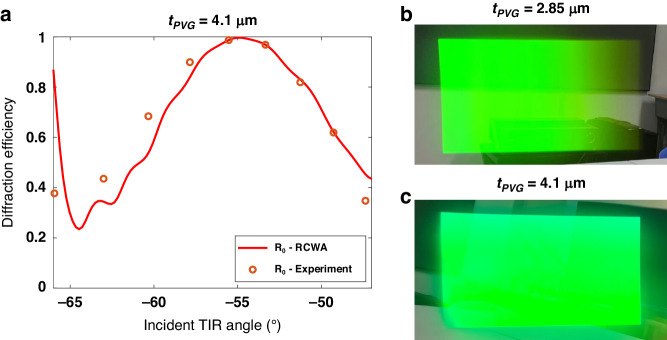
Experimental results of angular performance of the anomalous polarization conversion in PVG. **a** Angular response of 0th order (${R}_{0}$) diffraction efficiency during the second interaction for the incident RCP light with an extra TIR phase shift. Images of 20° FoV captured in a PVG-based waveguide display with the PVG thickness of (**b**) 2.85 µm and (**c**) 4.1 µm

While the polarization conversion phenomenon has been successfully demonstrated at normal incidence using a laser source, it is crucial to assess its angular performance. By varying the incident angle of the laser source, the angular performance of the second interaction is investigated, as shown in Fig. 4a, which agrees well with the RCWA simulation. Furthermore, an LCoS light engine with a diagonal FoV of 20° was employed to evaluate the angular performance of the polarization conversion in PVG-based waveguide displays. As Fig. 4b, c shows, the waveguide display with a 4.1 µm-thick PVG exhibits a much higher efficiency and better uniformity than that with a 2.85 µm-thick PVG.

Although the polarization conversion phenomenon in a PVG has been well verified using a laser source and an LCoS light engine, these experiments do not fully demonstrate its full potential due to the limited birefringence (${\Delta }n$) and FoV. Therefore, in the following, we will use a reactive mesogen with ${\Delta }n=0.4$ to thoroughly analyze the potential of the polarization conversion phenomenon in waveguide-based AR displays with a large FoV of 50°.

**Fig. 5:**
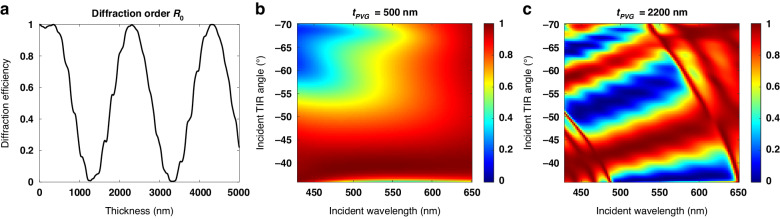
Response of multiple half-wave polarization conversion in PVG. **a** Reflected 0th order diffraction efficiency varying with the PVG thickness. Spectral and angular response of reflected 0th order at PVG thickness of (**b**) 500 nm (first-order half-wave condition) and (**c**) 2200 nm (second-order half-wave condition). The birefringence of PVG for simulation is 0.4 ($${n}_{e}=2.0,\,{n}_{o}=1.6$$); input and output media are respectively glass (*n*_*g*_ = 1.7$$)$$ and air; horizontal period $$\left({\Lambda }_{x}\right)$$ and slanted angle ($$\alpha )$$ of the PVG are 407 nm and 23.3°; the incident angle $$(\theta )$$ and wavelength $$(\lambda )$$ are −43° and 532 nm

It should be noted that, similar to a half-wave plate (HWP), the angular bandwidth of the polarization conversion is inherently limited due to dispersion. Additionally, multiple half-wave conditions exist, as depicted in Figs. 2e and 5a. Importantly, as the order of the half-wave condition increases, the angular bandwidth narrows further, as Fig. 5b, c depicts, making it impractical to cover the entire TIR region within the waveguide. However, it is important to note that the light loss typically increases as the TIR angle decreases, indicating that a significant light loss predominantly occurs on one side of the FoV. By satisfying the limited half-wave condition around the minimum TIR angles, a substantial improvement in in-coupling efficiency and uniformity can be achieved.

Next, we further investigate the angular performance of the first-order and second-order polarization conversions in a PVG-based waveguide display with 50° [30° (H) × 40° (V)] diagonal FoV at λ = 532 nm. Both orders demonstrate significant enhancements in in-coupling efficiency and uniformity throughout the entire FoV, surpassing the theoretical in-coupling efficiency limit of conventional diffractive in-couplers.

**Fig. 6:**
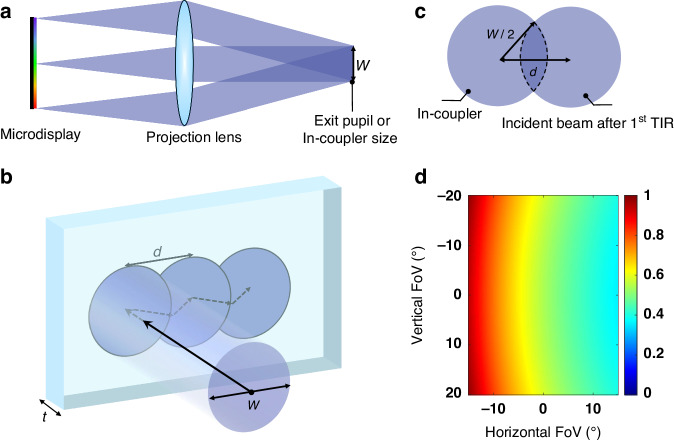
Design of light engine and waveguide combiner. **a** Light engine of waveguide displays. **b** Schematic of light propagation inside a waveguide around in-coupler region. **c** Cross section of the second interaction between in-coupler and one beam for a certain FoV. ${W}$ represents the diameter of the in-coupler or exit pupil size of light engine, t indicates the thickness of waveguide and $d$ represents the TIR propagation distance. **d** Theoretical in-coupling efficiency limit for the design with a FoV of 50° (30° (H) × 40° (V)) in Table 1

Before conducting polarization raytracing, the system configuration and parameters must be designed meticulously, including the display panel, collimation lens, in-coupler, and out-coupler. For the light engine, we assume that the in-coupler of the waveguide display (or exit pupil of the light engine) is a circle with a diameter $W$= 3 mm, which depends on the design of the collimation lens and the emission cone of the display panel, as depicted in Fig. 6(a). In most waveguide-based displays, the emission cone is typically very small, around ±15°. For our calculations, we employ an ideal lens as the collimation lens. Therefore, the focal length of the collimation lens (CL) can be calculated as follows:

5 $$f=W/\left(2\tan \left(15^{\circ} \right)\right)=5.598\,{\rm{mm}}$$

Moreover, to achieve a diagonal FoV of 50° [30° (H) × 40° (V)], the panel size is set to 3 mm × 4.075 mm. Subsequently, a waveguide substrate with thickness $t$ = 0.55 mm and index $n$ = 1.7 is utilized for design and simulation. Furthermore, to ensure a continuous eyebox, we assume that the pupil is continuous at the maximum TIR angle ${\theta }_{\max }$. This implies that each TIR propagation distance $d$ at the maximum TIR angle is equal to the in-coupler size $W$, as illustrated in Fig. 6b, c. Based on following equation:

6 $$d=2t* \tan {\theta }_{\max }=W$$

the maximum TIR angle is around 70°. Therefore, the minimum TIR angle ${\theta }_{\min }$ = 38° and the horizontal period ${\Lambda }_{x}\cong 407\, {\rm{nm}}$ of the in-coupler and out-coupler can be derived from the FoV = 50° [30° (H) × 40° (V)]. In summary, all the parameters of this waveguide system are listed in Table 1.

**Table 1:** Design parameters of light engine and polarization volume gratings in waveguide displays

Parameters	Design value
FoV	$50^{\circ}$ (30° (H) × 40° (V))
In-coupler size	3 mm
Working wavelength	532 nm
Panel Size	3 mm × 4.075 mm
Focal length of CL	5.6 mm
Refractive index of waveguide	1.7
Thickness of waveguide	0.55 mm
Maximum TIR angle	70°
Birefringence of PVG	0.4
Horizontal period of PVG	407 nm
Slanted angle of PVG	23°

Due to the multiple interactions between incident light and conventional in-couplers^12^, the theoretical in-coupling efficiency limit can be calculated based on the overlapping area ($${A}_{o})$$ of the second interaction between the incident beam and the in-coupler, as depicted in Fig. 6b, c. More specifically, the theoretical in-coupling efficiency limit (*E*) of a certain field can be expressed by the following equation:

7 $$E=1-\frac{{A}_{o}}{{A}_{{in}}}=1-\frac{2}{\pi }{\cos }^{-1}\left(\frac{d}{W}\right)+\frac{2d}{\pi {W}^{2}}\sqrt{{W}^{2}-{d}^{2}}$$

where ${A}_{{in}}$ is the area of in-coupler size or the exit pupil area of light engine. For instance, for the left-top corner FoV, it has a maximum in-coupling efficiency limit of 100% since there is no second interaction between the incident light and the in-coupler. As indicated in Fig. 6d, the minimum efficiency of the 50° FoV waveguide display is only around 36%, implying that only 36% of the in-coupling efficiency can be utilized to maintain good uniformity throughout the entire FoV.

**Fig. 7:**
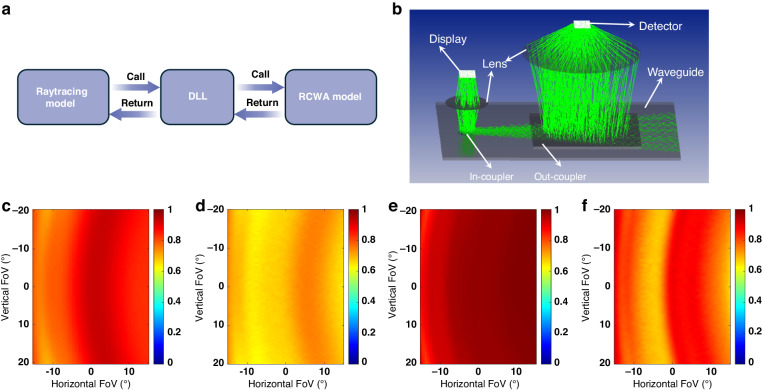
Polarization raytracing results of PVG as an in-coupler in waveguide displays. **a** Dynamics workflow between RCWA and Raytracing. **b** Shaded configuration of a waveguide display with a low-efficiency out-coupling grating. **c** Angular response of PVG with birefringence of 0.4 at slanted angle of 23° and thickness of 0.7 µm. **d** Improved in-coupling efficiency with optimized PVG by achieving the first-order half-wave condition. **e** Angular response of PVG with birefringence of 0.4 at slanted angle of 23.3° and thickness of 2.41 µm. **f** Improved in-coupling efficiency with optimized PVG by achieving the second-order half-wave condition

To analyze how to improve the in-coupling efficiency and uniformity using PVG as an in-coupler, we conduct polarization ray-tracing simulations using OpticStudio (Ansys Zemax). The RCWA model of PVG is compiled into a dynamic-link library (DLL) file and linked to OpticStudio, operating in non-sequential mode. As shown in Fig. 7a, during the ray tracing process in Zemax OpticStudio, if a ray hits the grating with a DLL, RCWA is automatically called to solve the field response and provide return data. The mathematical construction process is detailed in our previous research^26^. To measure the in-coupling efficiency, an ideal grating is used as the out-coupler to couple all the light out of the waveguide. At the same time, an ideal lens and detector are used to mimic the eye and detect the efficiency as shown in Fig. 7b. Additionally, an anti-reflection coating is applied to the waveguide substrate. To have a better understanding of the polarization conversion process, both first- and second-order half-wave conditions in Fig. 5b, c are studied in the following. First, through optimizing the PVG thickness and slanted angle to satisfy the first-order half-wave condition, the optimal efficiency and uniformity of the in-coupling process are achieved at 23° slanted angle and 0.7 μm thickness. The angular response of such a PVG is simulated by RCWA. As shown in Fig. 7c, the average diffraction efficiency is around 80%. Based on the polarization raytracing, the minimum in-coupling efficiency is improved from 36% to 61.3% (1.7× improvement), as shown in Fig. 6c. Besides, the uniformity within the entire FoV is improved from 36% to 85.9% (2.39× improvement) if the following definition of uniformity $U$ is adopted:

8 $$U=\frac{{I}_{\min }}{{I}_{\max }}* 100 \%$$

where ${I}_{\min }$ and ${I}_{\max }$ represent the minimum and maximum brightness through the whole FoV, respectively. If the in-coupling efficiency of conventional couplers is also considered as 80%, then the in-coupling efficiency and uniformity will be improved by ~2× and ~2.3×, respectively.

Furthermore, by optimizing the thickness and slanted angle of the in-coupler PVG to satisfy the second-order half-wave condition around the extreme field, the in-coupling efficiency and uniformity can be improved to 63.8% (1.77x enhancement) and 75.3% (2.09× enhancement), respectively, at slanted angle of 23.3° and thickness of 2.41 μm.

## Discussion

**Fig. 8:**
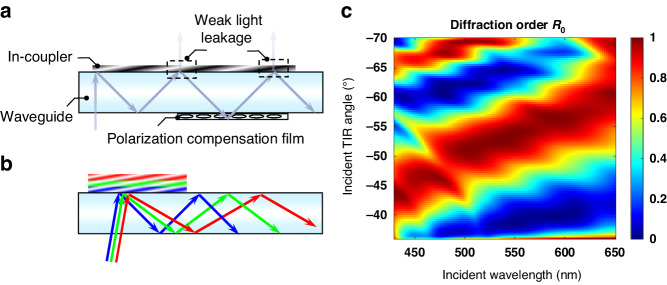
Generalization of the polarization conversion in PVG-based waveguide displays. **a** Polarization compensation layer in the in-coupling process^1^. **b** RGB lights propagating in a single waveguide. **c** Angular and spectral response of diffraction order ${R}_{0}$ with an optimized two-layer PVG as an in-coupler in a single waveguide for a full-color display

To further enhance the in-coupling efficiency and uniformity, one straightforward approach is to utilize a waveguide with a higher refractive index. However, it’s worth noting that the in-coupling efficiency may decrease again as the FoV gets wider. Besides, such polarization conversion in PVG can only address the efficiency issues caused by the second interaction between the incident beam and the in-coupler. To further enhance the in-coupling efficiency affected by the third interaction, as illustrated in Fig. 8a, an additional compensation layer can be incorporated at the in-coupler PVG. This would achieve polarization conversion over a wider angular response^1,11^ and further overcome the third interaction, resulting in nearly all light being coupled into the waveguide with an approximately threefold enhancement. However, this approach may impose significant challenges in terms of fabrication requirements for the waveguide display system.

Besides, our results can be readily applied to various waveguide designs, including different pupil expansion schemes and different numbers of waveguides^1^, for example, 1D EPE, 2D EPE, full-color display in a single waveguide, two waveguides, and three waveguides. Additionally, utilizing a multi-layer PVG will not only expand the angular and spectral responses but also enhance the in-coupling efficiency by satisfying the half-wave conditions for different incident angles and wavelengths, especially for full-color displays in a single waveguide. Clearly, satisfying the half-wave condition for RGB lights in three separate waveguides is feasible because each waveguide exclusively transmits a single color, requiring each in-coupler to meet the half-wave condition for that specific color. It is feasible even when using LED-based light engines with a wide spectrum (e.g., 40 nm full width at half maximum), because the spectral bandwidth at the first-order half-wave condition is sufficiently wide, as Fig. 5b shows. However, achieving full-color display with a single waveguide necessitates simultaneous satisfaction of the half-wave conditions for RGB lights, posing challenges for a single-layer PVG. Additionally, the half-wave condition must be met at different TIR angles for RGB lights due to the dispersion of the in-coupler, as illustrated in Fig. 8b. Specifically, same incident angles of RGB lights will be diffracted to various TIR angles based on the following diffraction equation:

9 $${n}_{{in}}\sin \left({\theta }_{{in}}\right)+\frac{\lambda }{{\Lambda }_{x}}={n}_{{out}}\sin \left({\theta }_{{out}}\right)$$

where ${n}_{{in}}$ and ${n}_{{out}}$ are the refractive indices of the input and output media, $\lambda$ represents the incident wavelength, and ${\theta }_{{in}}$ and ${\theta }_{{out}}$ are the incident and diffracted (TIR) angles, respectively. It is evident that the diffracted (TIR) angle increases with the increase in wavelength for the same incident angle. Following this principle, a two-layer PVG^27^ with a birefringence $\varDelta n$ = 0.4 is meticulously optimized to satisfy the half-wave conditions for RGB lights at varying TIR angles. Illustrated in Fig. 8c, the optimized angular and spectral response of the diffracted ${R}_{0}$ order is achieved with the first-layer thickness of 820 nm and the second-layer thickness of 900 nm. The designed slant angle for the two layers is 21.2° and 25.6°, respectively, with a horizontal period of approximately 412 nm, while the index of the waveguide substrate is 1.7. Consequently, the polarization conversion phenomenon in the multi-layer PVG helps enhance the in-coupling efficiency and uniformity throughout the FoV for a full-color display.

More importantly, the polarization properties will facilitate more efficient rolling *k* vector designs^28^ and laser-based waveguide designs^1^. In these two designs, the beams from different FoVs do not overlap at the in-coupler. This implies that the in-coupler at different spatial positions can be locally modulated to adjust the half-wave conditions for different incident angles. Such in-coupler could be easily fabricated with inkjet printing^29^.

While the polarization conversion phenomenon in PVG can significantly enhance the in-coupling efficiency and uniformity, its efficacy is heavily dependent on the polarized light sources. When the light source is polarized, such as in a Liquid-Crystal-on-Silicon (LCOS) panel, PVG with the novel polarization properties demonstrates substantial advantages over conventional in-couplers. However, when using an unpolarized light source like micro-LEDs, a single PVG with the novel polarization property may only achieve a comparable level to traditional polarization-independent in-couplers due to the polarization selectivity of CLC. Nonetheless, this polarization selectivity can be leveraged to implement polarization multiplexing in two waveguides^30^ utilizing different circular polarization-dependent PVGs with an unpolarized light source. Consequently, PVG would still exhibit a superior in-coupling efficiency and uniformity in such scenarios.

As the polarization conversion phenomenon is intricately linked to the PVG thickness, careful consideration of PVG surface roughness during the fabrication process is essential. In Fig. 3b, we find that there exists a window where the polarization conversion is relatively insensitive to PVG thickness variations. However, as the LC birefringence increases, the polarization conversion becomes increasingly dependent on the PVG thickness. Based on the reported surface roughness of PVG^31^, the variation of PVG thickness can be well controlled to be below 25 nm, which barely affects the polarization conversion phenomenon. Besides, alternative fabrication methods, such as inkjet printing^29^ could potentially improve the PVG surface flatness.

Moreover, the fabrication procedures and complexity remain consistent with previous PVG iterations. Therefore, implementing the polarization conversion phenomenon incurs no additional cost, as it is inherent to PVG and was first identified in this study. However, compared to mature fabrication techniques^1,32,33^ (e.g., nanoimprinting lithography or ion beam etching) for SRGs, large-scale fabrication capability is imperative for future scalability to enable widespread applications of PVG.

In conclusion, we have discovered and demonstrated an anomalous polarization conversion phenomenon in PVGs. This new property effectively resolves the tradeoff between in-coupling efficiency and uniformity throughout the eyebox and FoV. By studying the multiple half-wave conditions in a PVG, we achieve a remarkable 2× improvement in in-coupling efficiency and 2.3× enhancement in uniformity across the FoV for a waveguide display with 50° FoV, compared to conventional couplers. To further overcome the in-coupling efficiency limit affected by the third interaction and achieve an approximately threefold enhancement, an additional compensation layer can be incorporated at the in-coupler PVG. Moreover, we delve into the broad applicability of the polarization conversion process, emphasizing its potential to be integrated into various waveguide display designs, especially full-color displays. Additionally, we examine and discuss the impact of the surface roughness of the PVG on the polarization conversion process. Overall, this polarization conversion phenomenon serves as the first evidence to showcase the superiority of PVG in-coupler in waveguide-based AR displays compared to other couplers. This advancement is expected to accelerate the development of high-efficiency waveguide-based AR displays and contribute to the commercialization of PVG technology.

## Material and methods

### Materials

The photoalignment material used in our experiments is Brilliant Yellow (BY) from Sinopharm Chemical Reagent Co., Ltd. BY powders were dissolved in dimethyl-formamide with a weight concentration of 0.5%. The mixed solution was filtered using a 0.2 μm Teflon syringe before spin-coating onto the glass substrate. The LC mixture is composed of solvent toluene and precursor which contains LC monomer RM 257 purchased from Jiangsu Hecheng Advanced Materials Co., Ltd., surfactant Zonyl 8857A from Dupont, and photo-initiator Irgcure 184 from MACKLIN.

### Methods

**Fig. 9:**
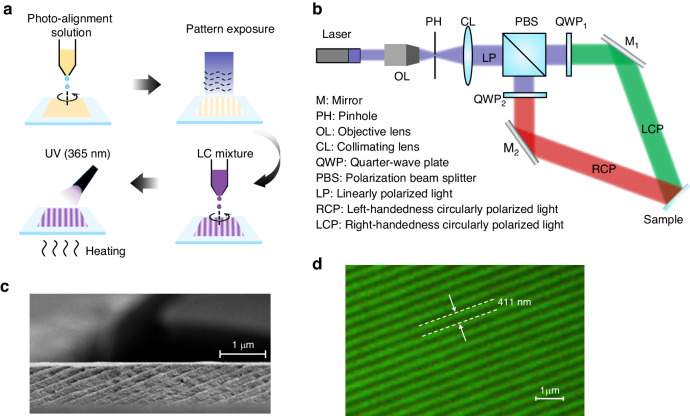
Fabrication of PVG. **a** Fabrication flowchart of PVGs. **b** Exposure setup for PVGs. **c** Cross section SEM image and (**d**) POM image of a PVG with a horizontal period of 411 nm

Figure 9a depicts the fabrication process of PVGs. Initially, the photoalignment material was spin-coated onto a clean glass substrate with hydrophilic treatment by plasma etching. Brilliant yellow (BY) was used as the photoalignment material and dissolved in N,N-Dimethylformamide (DMF) at a concentration of 0.5 wt%. Subsequently, the sample with the photoalignment film underwent polarized interference exposure, as shown in Fig. 9b. The expanded and collimated laser beam was split into two arms by a polarization beam splitter (PBS). Each beam was then converted to the opposite circular polarization using a quarter-wave plate (QWP), respectively. In our experiment, a 460 nm laser was employed as the recording beam, and the exposure angle was set at 34°. RM257 was utilized to create uniform LC layers after spin-coating. Finally, the PVGs were exposed to UV light for stabilization. In accordance with the predetermined specifications, PVGs were fabricated, and the structures are shown in Fig. 9c through a polarizing optical microscope (POM) and in Fig. 9d through a cross-section scanning electron microscope (SEM).

The 0.7 mm-thick glass substrates were purchased from Luoyang Guluo Glass. The substrate was cleaned using ethanol and then treated by vacuum plasma for 40 s before the spin-coating of BY solutions. The humidity of the environment for spin-coating was controlled to be under 40%. The BY layer on the glass substrate was exposed to a 460 nm laser (Coherent, Genesis CX-460) with 1 W output power for 2 min. We preheated the LC mixture on a hot plate stage at 70 °C before spin-coating because the viscosity decreases with increased temperature. Besides, we also put the LC substrates on top of the hot plate right after the spin-coating process for several seconds to obtain better alignment. Detailed recipes are summarized in Table 2. The PVG thickness was measured with a profiler from BRUKER. In Fig. 3, an out-coupling prism is used to measure the diffraction efficiency (${R}_{0}$) at the second interaction with a laser source featuring a small beam size. This deliberate choice facilitates the clear differentiation and measurement of multiple beams post-interaction with the PVG through a power meter. To ensure the incident light to the PVG is circularly polarized, a circular polarizer is inserted after the laser source. Additionally, to verify the measurement accuracy, a reference point (surface reflection of glass substrate) is established using a clean glass substrate without PVG.

**Table 2:** Materials and coating speed for PVGs fabrication

Sample	Solute	Solvent	Concentration	Coating speed (rpm)	Sample thickness (nm)
1	RM257 (95.06%) R5011 (2.09%) Irgcure 184(2.85%)	Toluene	6 wt%	600 (30 s)	500
2	–	–	12 wt.%	1500 (30 s)	720
3	–	–	12 wt%	1000 (30 s)	960
4	–	–	12 wt%	600 (30 s)	1200
5	–	–	18 wt%	1500 (30 s)	1650
6	–	–	38 wt%	3000 (30 s)	2850
7	–	–	38 wt%	2500 (30 s)	3050
8	–	–	38 wt%	2000 (30 s)	3300
9	–	–	38 wt%	1000 (30 s)	4400

## Data Availability

All data needed to evaluate the conclusions in the paper are present in the paper. Additional data related to this paper may be requested from the authors.
